# Hydrogen-Peroxide Synthesis and LDL-Uptake Controls Immunosuppressive Properties in Monocyte-Derived Dendritic Cells

**DOI:** 10.3390/cancers13030461

**Published:** 2021-01-26

**Authors:** Ann-Katrin Menzner, Tanja Rottmar, Simon Voelkl, Jacobus J. Bosch, Dimitrios Mougiakakos, Andreas Mackensen, Yazid J. Resheq

**Affiliations:** 1 Department of Internal Medicine 5, Hematology/Oncology, Friedrich Alexander University Erlangen Nuremberg, Ulmenweg 18, 91054 Erlangen, Germany; ak.menzner@googlemail.com (A.-K.M.); tanja.rottmar@fau.de (T.R.); simon.voelkl@uk-erlangen.de (S.V.); bosch.jacobus@mh-hannover.de (J.J.B.); dimitrios.mougiakakos@uk-erlangen.de (D.M.); andreas.mackensen@uk-erlangen.de (A.M.); 2 Clinical Research Center Hannover, MH Hannover, Feodor-Lynen-Straße 15, 30625 Hannover, Germany

**Keywords:** tolerogenic dendritic cells, catalase, hydrogen peroxide, reactive oxygen species, tumor immune escape, LDL-uptake, IDO

## Abstract

**Simple Summary:**

Given the encouraging success of immunotherapy in cancer, the role of metabolism in tumor immune-evasion is an emerging research field with a unique potential to overcome current limitations in immunotherapy. Herein, hepatic stromal cells, which may act as immunological bystanders in cancer, are capable of inducing immunosuppressive phenotypes in monocytic cells by controlling hydrogen peroxide (H_2_O_2_-) metabolism. As monocytic cells play an important role in tumor-immunology, we sought to identify the underlying mechanisms. Herein, we unraveled a complex interaction between cholesterol/LDL- and H_2_O_2_-metabolism: Extracellular depletion of H_2_O_2_ leads to enhanced H_2_O_2_-production with a consecutive increase in LDL-uptake throughout differentiation of monocytes to monocyte-derived dendritic cells and, as a result, to the induction of distinct immunosuppressive properties. These findings shed new light on the role of LDL-metabolism in tumor-immunology and might help to further improve immunotherapeutic approaches against cancer.

**Abstract:**

Background and Aims: Induction of myeloid-derived suppressor cells (MDSC) is a critical step in immune cell evasion by different cancer types, including liver cancer. In the liver, hepatic stromal cells orchestrate induction of MDSCs, employing a mechanism dependent on hydrogen peroxide (H_2_O_2_) depletion. However, the effects on monocyte-derived dendritic cells (moDCs) are unknown. Methods: Monocytes from healthy donors were differentiated to moDCs in the presence of extracellular enzymatic H_2_O_2_-depletion (hereinafter CAT-DCs), and studied phenotypically and functionally. To elucidate the underlying molecular mechanisms, we analyzed H_2_O_2_- and LDL-metabolism as they are interconnected in monocyte-driven phagocytosis. Results: CAT-DCs were of an immature DC phenotype, particularly characterized by impaired expression of the costimulatory molecules CD80/86. Moreover, CAT-DCs were able to suppress T-cells using indoleamine 2,3-dioxygenase (IDO), and induced IL10/IL17-secreting T-cells—a subtype reported to exert immunosuppression in acute myeloid leukemia. CAT-DCs also displayed significantly increased NADPH-oxidase-driven H_2_O_2_-production, enhancing low-density lipoprotein (LDL)-uptake. Blocking LDL-uptake restored maturation, and attenuated the immunosuppressive properties of CAT-DCs. Discussion: Here, we report a novel axis between H_2_O_2_- and LDL-metabolism controlling tolerogenic properties in moDCs. Given that moDCs are pivotal in tumor-rejection, and lipid-accumulation is associated with tumor-immune-escape, LDL-metabolism appears to play an important role in tumor-immunology.

## 1. Introduction

Malignant tumors are capable of evading immune response, in a mechanism known as tumor-immune-escape [1,2]. Putative mechanisms include the induction of myeloid-derived suppressor cells (MDSCs) [3], tolerogenic dendritic cells (DCs) [4], and regulatory T-cells (T_regs_) [5]. This induction either happens by enhanced production of specific cytokines [6,7], or indirectly by employing bystander cells, most notably stromal cells [8,9]. In this respect, monocyte-derived DCs (moDCs) represent a subset with distinct properties allowing them to accumulate in inflamed tissue [10,11]. Conversely, within the tumor-microenvironment moDCs gain tolerogenic properties, and are capable of suppressing T-cell-proliferation by indoldiaminoxidase (IDO)-driven tryptophan-depletion, or by the induction of T_reg_ [12]_._

Monocytic cells are dependent on reactive oxygen species (ROS) for their phagocytic activity, most notably hydrogen-peroxide (H_2_O_2_) [13]. Tumor cells are capable of producing ROS in substantial quantities due to altered metabolism (i.e., enhanced oxidative phosphorylation), which has been shown to contribute to tumor-immune-escape [14,15]. In turn, tumors employ various mechanisms to detoxify ROS, as the increased accumulation of these metabolites may have detrimental effects [16,17].

Despite encouraging results from experimental studies [18,19], detoxifying ROS has not been successful as a cancer therapy in vivo [20]. A potential explanation might be that ROS play an equally ambiguous role in immune homeostasis. Indeed, we have shown that hepatic stromal cells are capable of inducing MDSCs from human monocytes by contact-dependent depletion of H_2_O_2_ [21]. This led us to study metabolic interactions between stromal cells and monocytes, and the role of ROS in cellular differentiation more broadly. More specifically, we sought to analyze the impact of H_2_O_2_-depletion throughout the differentiation and maturation of this cellular lineage. In so doing, we uncovered a previously unreported link between H_2_O_2,_ LDL metabolism and immune-modulation in moDCs.

## 2. Materials and Methods

### 2.1. Reagents

All cells were cultured in RPM-1640 medium containing 10% FCS, 1% Penicillin/Streptomycin and 2 mM L-glutamine. Untouched T-cell isolation Kit, Human T-Activator CD3/CD28 Dynabeads, Amplex Red Reagent, fixable viability dye SYTOX blue nucleic acid stain, NP40 Cell Lysis Buffer and Horseradish Peroxidase were purchased from Thermo Fisher (Waltham, MA, USA). Catalase, glutathione peroxidase, 1-methyl tryptophan (1-MT), dimethyl sulfoxide (DMSO), phorbol 12-myristate 13-acetate (PMA), ionomycin and paraformaldehyde (PFA) were purchased from Sigma-Aldrich (St. Louis, MO, USA). Lymphoprep gradient was purchased from Progen Biotechnik (Heidelberg, Germany). Microbeads for CD14, CD4 and CD8 cell isolation as well as LS isolation columns were purchased from Miltenyi Biotec (Bergisch Gladbach, Germany). Tag-it Violet Proliferation and Cell Tracking Dye was purchased from Biolegend (San Diego, CA, USA). IL10R monoclonal antibody (mAb) was purchased from Acris Abs (San Diego, CA, USA). NS-398 was purchased from Cayman Chemicals (Ann Arbor, MI, USA). Proteome Profiler Human Cytokine Array Kit/ARY005 was purchased from R&D systems (Minneapolis, MN, USA). BD Cytofix/Cytoperm Kit and GolgiStop were purchased from Becton Dickinson (Franklin Lakes, NJ, USA). VersaComp Antibody Capture Beads were obtained from Beckman Coulter (Brea, CA, USA). Tissue-culture plastic was obtained from Becton Dickinson (Franklin Lakes, NJ, USA), Nunc (Roskilde, Denmark), and Sarstedt (Nuembrecht, Germany). A complete list of antibodies and Ab-clones used can be viewed in Table S1.

### 2.2. Isolation of Mononuclear Cells from Leukapheresis Concentrates

Human leukapheresis concentrates from Leukoreduction System (LRS) chambers were obtained from the Department of Transfusion Medicine and Haemostaseology (University Hospital Erlangen, Erlangen, Germany) after ethics committee approval and informed patient consent was received (Ethics Committee reference number 36_12 B). Peripheral blood mononuclear cells (PBMCs) were isolated using density gradient centrifugation over Lymphoprep gradient (20 min. at 800× *g*). CD14^+^ monocytes, CD4^+^ and CD8^+^ T-cells were enriched by positive selection using MACS LS-columns and beads according to the manufacturer’s recommendations (Miltenyi Biotec, Bergisch Gladbach, Germany). Untouched PAN T-cells were enriched by negative selection using a biotin-conjugated antibody cocktail which contains antibodies against CD14, CD15, CD16, CD19, CD34, CD36, CD56, CD123, and CD235a (Glycophorin A) according to the manufacturer’s recommendations (Dynabeads, Thermo Fisher). Flow cytometric control of monocytes and T-cells showed ≥95% purity for all experiments.

### 2.3. Generation of Dendritic Cells from Human Monocytes

Magnetically enriched human CD14^+^ monocytes were seeded at 1 × 10^6^ cells/mL in 24 well flat-bottom plates after viability was confirmed via trypan blue exclusion. All cells were cultured in medium at 37 °C, 5% CO_2_. Monocytes were differentiated to mature DCs as described previously [22]. In brief, ex vivo monocytes were incubated in medium containing 500 IU/mL IL4 and 500 IU/mL GM-CSF for 5 days. For final DC maturation, 1 µg/mL LPS was added on day 5 for 48 h. Mature dendritic cells (mDCs) were defined as CD11c^+^, CD80^+^/CD86^+^ and CD14.

For depletion of H_2_O_2_, catalase was introduced to monocyte-cultures at a concentration of 1000 U/mL during differentiation and maturation (CAT-DCs), followed by washing-step (2×) in order to remove any residual catalase. In an additional “proof-of-concept” experiment, the alternative hydrogen-peroxide scavenger glutathione peroxidase (GPX) was added at 250 U/mL instead of catalase. DC maturation was assessed using flow cytometry. Appropriate catalase and GPX concentrations were determined in preliminary experiments.

For inhibition of LDL receptor (LDL-R), LRP1 and CD36 monoclonal antibodies were used at the following concentrations: 5, 10 and 15 µg/mL. Inhibition of NADPH oxidase was achieved using DPI (Diphenyleneiodonium, Sigma Aldrich) at a concentration of 10 µM. For selective depletion of mitochondrial H_2_O_2_, 20 nM MitoQ (10-(6′-ubiquinolyl)decyltriphenylphosphonium) was added as previously described [23].

### 2.4. Coculture of DCs and T-Cells

To determine the suppressive capacity of induced CAT-DCs, 50,000 allogeneic PAN T-cells were labeled with Tag-it Violet Proliferation and Cell Tracking Dye, and cocultured in different ratios with DCs or CAT-DCs (1:0, 1:1, 2:1, 4:1, and 8:1) in 96 well plates for 5 days in the presence or absence of Human T-Activator CD3/CD28 dyna beads according to the manufacturer’s recommendations. T-cell proliferation was analyzed by Cell Tracking Dye dilution in successive cell divisions as measured by flow cytometry. The role of distinct immunosuppressive mechanisms was assessed by the introduction of blocking antibodies (IL-10R) and inhibitors (cyclooxygenase-2 [COX-2] and IDO). For blocking and inhibition experiments, concentrations which have been reported to be effective in preventing T-cell suppression were used [21]: 5 mg/mL IL-10R blocking Abs, 20 mM NS398, 500 mM 1-methyl-DL-tryptophan (1-MT).

To study the effects of CAT-DCs on T-cells, PAN T-cells, CD4^+^ or CD8^+^ T-cells were cocultured with DCs or CAT-DCs in 24 well plates at a ratio of 4:1 for 5 days with or without Human T-Activator CD3/CD28 stimulation.

### 2.5. Measurement of H_2_O_2_ and Superoxide Levels

H_2_O_2_-levels in supernatant and cell lysates of day 7 DCs and CAT-DCs were quantified using the AMPLEX Ultra Red reagent. Samples were incubated with 200 mM AMPLEX Ultra Red reagent and 2 U/mL HRP. Detection of the fluorescent product resorufin was measured in a Spectra Max M3 plate reader (Molecular Devices, San Jose, CA, USA) with excitation and emission wavelengths of 530 and 590 nm, respectively (slit widths 25/35 nm). Mitochondrial superoxide was analyzed by “MitoSOX Red mitochondrial superoxide indicator” (Thermo Fisher) according to the manufacturer’s protocol and analyzed by flow cytometry.

### 2.6. Proteome Profiling

Semi-quantitative immunosorbent assessment of cytokine expression was performed as described before [21]. In brief, DCs and CAT-DCs were harvested on day 7 and lysed using NP-40 Cell Lysis Buffer. Assays were performed according to the manufacturer’s instructions, luminescence measured using an Amersham Imager 680 (GE Healthcare, Chicago, IL, USA), and relative luminescent units (RLU) quantified using ImageJ (NIH, Bethesda, MD, USA) [24].

### 2.7. Flow Cytometry

For surface marker staining, cells were harvested, washed using staining buffer (PBS, 2% FCS and 2 mM EDTA) and collected by centrifugation. Cells were stained using fluorochrome-conjugated monoclonal antibodies [25] followed by incubation for 20 min at 4 °C, two washing steps, and resuspension in staining buffer. Viable cells were selected by Sytox blue exclusion with confirmed viability of >95%. For intracellular staining, cells were fixed and permeabilized using BD Cytofix/Cytoperm Kit. For cytokine staining, cells were stimulated with PMA (20 ng/mL) and ionomycin (1 μg/mL) and treated with 4 µL BD GolgiStop for 4 h before staining. Compensation was performed using antibody capture beads. The gating strategy was set as follows: FSC/SSC to determine monocytic or T-cell populations, FSC-A/FSC-H to gate out doublets, live gate (SYTOX blue negative, which represents the fraction of viable cells within the sample) and either CD80-PE-Cy7/CD86-APC for DC analysis or CD14-PerCP-Cy5.5/CD3-BUV737 and CD4-BUV395/CD8-BUV496 for T-cell analysis. A detailed list of antibodies is provided in Table S1. Acquisition and analysis was carried out using a BDCanto II or BD LSR Fortessa cytometer (Becton Dickinson) and Kaluza Analysis software (Beckman Coulter).

### 2.8. LDL and oxLDL-Uptake Assay

To measure the uptake of LDL (“low density protein”) and oxLDL (“oxidized LDL”), mDCs and CAT-DCs were incubated with phRodo RED-LDL or dil-oxLDL reagent, respectively (both Thermo Fisher), according to the manufacturer’s recommendations. Fluorescence was measured by flow cytometry using a BDCanto II cytometer (Becton Dickinson).

### 2.9. Statistical Analysis

Normally distributed continuous variables were expressed as mean (SD), and continuous variables with skewed distribution were expressed as median (range). Differences in means, medians, and correlation analyses were evaluated with parametric and nonparametric tests (paired two-tail Student’s *t*-test and ANOVA calculation). All statistical analyses were performed using GraphPad Prism Version 5 (GraphPad Prism Software, San Diego, CA, USA). A *p*-value < 0.05 was considered significant.

## 3. Results

### 3.1. Catalase-Mediated H_2_O_2_ Depletion Impairs Maturation of Monocyte-Derived DCs

**Figure 1:**
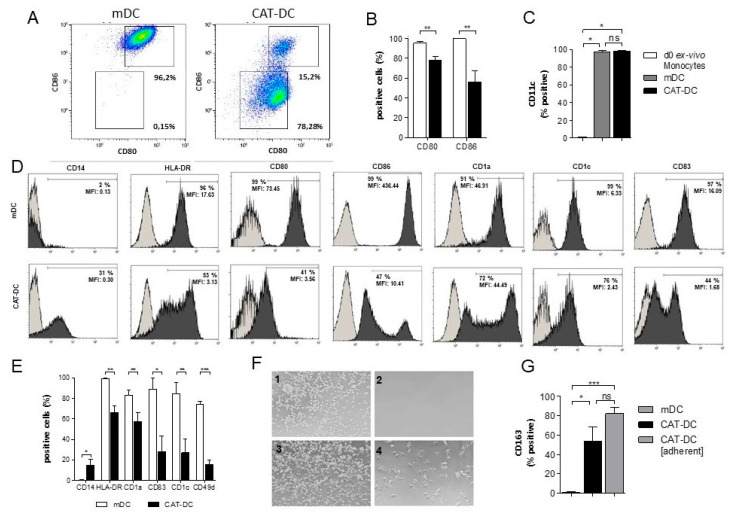
Differentiation and maturation of human moDCs in the presence of catalase leads to an immature phenotype. Analysis of surface marker expression by flow cytometry. Monocytes differentiated and matured in the presence of IL4, GM-CSF and LPS were defined as mDCs and characterized as CD80^+^/CD86^+^. moDCs additionally treated with purified catalase during differentiation and maturation where defined as CAT-DCs. (**A**,**B**) Flow-cytometric analysis of CD80 and CD86 expression of CAT-DCs compared to mDCs (A: representative plots; B: mean % values from *n* = 6 experiments with SD values). (**C**) Flow-cytometric analysis of CD11c on ex-vivo monocytes, mDCS and CAT-DCs. (**D**) Exemplary histograms of cell surface markers expressed on mDCs and CAT-DCs after 7 days of culture as analyzed by flow cytometry. Gray plots: isotype controls; MFIs represents the MFIs of all cells analyzed by flow cytometry for the according surface marker (raw data can be found in Figure S3). (**E**) Flow-cytometric analysis of maturation-marker-expression on CAT-DCs as compared to mDCs. (**F**) Formation of an adherent, morphologically distinct subpopulation in DCs differentiated and matured in the presence of catalase; exemplary images shown for mDCs (1) and CAT-DCs (3) after 7 days of culture vs. mDCs (2) and CAT-DCs (4) culture dishes after gentle detachment by washing. (**G**) Comparison of CD163 expression of mDCs, non-adherent CAT-DCs, and adherent CAT-DCs (*n* = 3). General gating strategy for flow-cytometric analysis of DC surface markers with dead cell exclusion using SYTOX blue dead cell stain and definition of mDCs as CD80^+^/CD86^+^ and CAT-DCs as CD80^low/−^/CD86^−^ can be viewed in Figure S1. Statistical analysis was performed with a paired two-tail Student’s *t*-test. *p*-values indicated as * *p* < 0.05, ** *p* < 0.01, *** *p* < 0.001.

We have recently shown that hepatic stellate cells employ a catalase-driven mechanism to induce monocytic MDSCs [26]. Given earlier studies indicating a critical role of H_2_O_2_ for monocytic cells, we speculated that H_2_O_2_ may serve as key-regulator in the differentiation/maturation of monocyte-derived APCs. First, monocytes were differentiated and matured to monocyte-derived DCs in the presence of catalase (herein after “CAT-DCs”). Adjunct, we analyzed them for the expression of distinct maturation markers by flow cytometry. CAT-DCs showed significantly reduced expression of the costimulatory molecules CD80 and CD86 in comparison to normally differentiated mature monocyte-derived DCs (hereinafter “mDCs”) (Figure 1A,B). While mDCs and CAT-DCs expressed the DC marker CD11c to the same extend (Figure 1C), expression of typical maturation markers such as CD1a, CD1c, CD83, and HLA-DR (MHC-II) was significantly reduced. Additionally, downregulation of CD14, which is typical for maturation of DCs, was impaired (Figure 1D,E, raw data Figure S3). No significant differences were observed in the expression of surface markers CD11b, CD33, CD40 and HLA-ABC (MHC-I) (Figure S2).

When maturing monocytes to DCs in the presence of catalase we noticed a change in morphology and adhesive properties (Figure 1F). Untreated mDCs were easily detachable after 7 days in culture (Figure 1(F1): pre-wash, Figure 1(F2): post-wash), but we observed a morphologically distinct, adherent subpopulation that could only be detached by firm washing when DCs were differentiated and matured in the presence of catalase (CAT-DCs) (Figure 1(F3): pre-wash, (F4): post-wash). Interestingly, 54% (*p* < 0.05) of normally detached CAT-DCs and 82% (*p* < 0.001) of adherent CAT-DCs expressed CD163 (Figure 1G), a surface marker indicative of M2-macrophages [27]. All other surface markers analyzed were unaffected in comparison to non-adherent CAT-DCs (Figure S2).

### 3.2. CAT-DCs Are Capable of Suppressing Pan-T-Cell Proliferation

**Figure 2:**
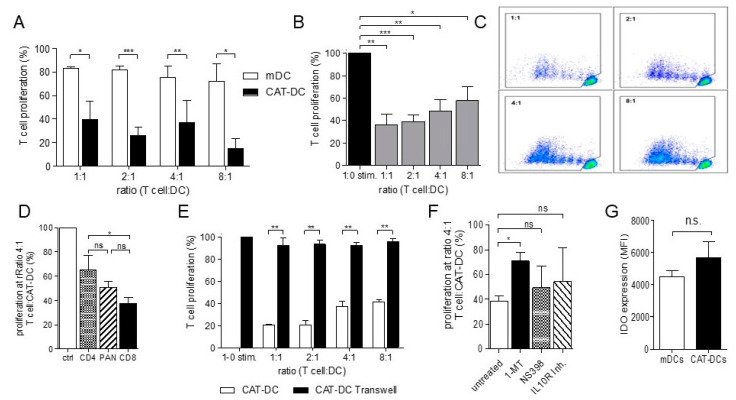
CAT-DCs suppress T-cell proliferation in a contact dependent, IDO-mediated manner. (**A**) Analysis of stimulatory capacity of CAT-DCs on PAN T-cells in comparison to mDCs after 5 days of coculture without addition of CD3/28-beads (*n* = 6). (**B**) Proliferation of CD3/CD28-stimulated T-cells cocultured with CAT-DCs for 5 days. Proliferation of T-cells in CAT-DC-cocultures are shown relative to stimulated control (*n* = 6). (**C**) Representative flow cytometry plots of T-cell proliferation following CD3/CD28-stimulation after 5 days of CAT-DC coculture. T-cells were stained with Tag-it Violet Proliferation and Cell Tracking Dye to visualize proliferation. (**D**) Comparison of proliferation of CD8^+^ T-cells, CD4^+^ T-cells, and CD3^+^ PAN-T-cells in coculture with CAT-DCs at a ratio of 4:1 T-cell:CAT-DC following CD3/CD28-stimulation (*n* = 3, representative plots: Figure S4C; General gating strategy for T-cell-analysis can be found in Figure S5). (**E**) Analysis of CD3/28-bead stimulated T-cell-proliferation applying physical separation (transwell inserts) throughout coculture of T-cells with CAT-DCs in decreasing ratios (*n* = 3). (**G**) Analysis of proliferation in cocultures of CD3/28-bead stimulated T-cells with CAT-DCs following introduction of indoleamine 2,3-dioxygenase inhibitor 1-MT, COX-inhibitor, and IL10-R blockade (**F**). Flow-cytometric analysis of intracellular IDO-expression in CAT-DCs as compared to mDCs (*n* = 3). Statistical analysis was performed with a paired two-tail Student’s *t*-test. (* *p* < 0.05, ** *p* < 0.01, *** *p* < 0.001).

Next, we investigated whether the effects of catalase are of functional relevance, by comparing the immune-stimulatory capacity of CAT-DCs and mDCs in coculture with allogenic Pan-T-cells. In so doing, CAT-DCs showed 5-fold reduced capacity to stimulate T-cells in vitro compared to untreated mDCs in the absence of CD3/CD28-stimulation (Figure 2A).

We then examined whether altered immunosuppressive properties in CAT DCs relate to altered expression of costimulatory molecules CD80 and CD86. Herein, allogenic, CD3/CD28-stimulated Pan-T-cells were cocultured with CAT-DCs in increasing ratios for 5 days. Significant suppression of T-cell proliferation was observed up to a ratio of 8 T-cells per CAT-DC, proving CAT-DCs to be functional in regulating T-cell responses (Figure 2B,C). Notably, CD8^+^ T-cells were more susceptible to CAT-DC-mediated suppression of CD3/CD28-triggered proliferation than CD4^+^ T-cells (37% to 64% at ratio of 4:1 T-cell:DC, *p* < 0.05; Figure 2D). In order to exclude that catalase, which might not have come off CAT-DCs throughout the washing process, may equally affect T-cell-proliferation, we cocultured CD3/28-activated T-cells with purified catalase at the same concentration as for induction of CAT-DCs. Herein, we observed that addition of catalase did not affect T-cell-proliferation (Figure S4).

### 3.3. CAT-DCs Suppress T-Cell Proliferation through a Contact-Dependent, IDO-Mediated Mechanism

To determine whether the mechanism of T-cell suppression by CAT-DCs is contact- dependent, we separated T-cells and CAT-DCs using non-cell-permissive transwell-inserts. Using this approach, significantly increased proliferation was observed when T-cells were separated from CAT-DCs (Figure 2E).

Recently, we reported that Catalase-induced MDSCs suppress T-cell-proliferation in an IDO-dependent manner [21]. As tolerogenic DCs found in the tumor-microenvironment employ similar mechanisms, we then analyzed if this holds true for CAT-DCs. Indeed, the IDO inhibitor 1-MT restored proliferative capacity of T-cells in coculture with CAT-DCs (38.30% to 70.97%, *p* < 0.05; Figure 2F); whereas COX-2-inhibition (NS398) and IL10R-inhibiting mAbs [28,29] had no significant effect on T-cell-proliferation. To further validate the role of IDO in CAT-DC-driven T-cell suppression, we analyzed CAT-DCs for IDO-expression using intracellular flow cytometry. Herein, CAT-DCs showed a trend to higher intracellular levels of IDO as compared to mDCs, although the difference did not reach significance (Figure 2G).

### 3.4. CAT-DCs Skew T-Cell-Polarization towards an Immunosuppressive Phenotype

**Figure 3:**
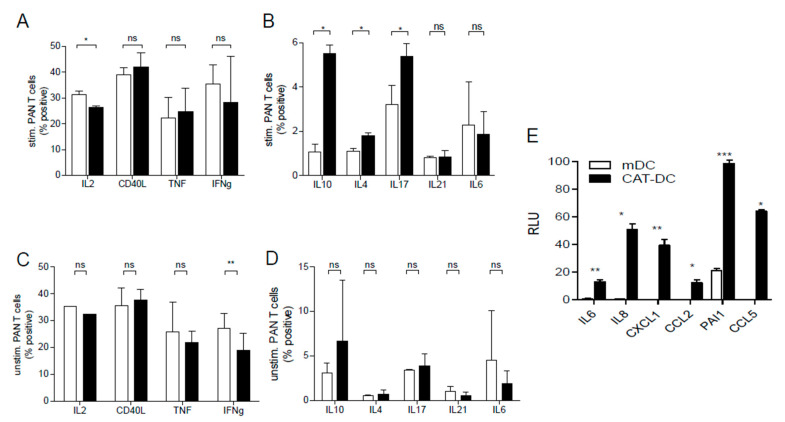
CAT-DCs induce changes in T-cell-polarization after 6 days of coculture and exhibit an altered cytokine profile. (**A**,**B**) Analysis of expression of IL2, CD40L, TNFα, IFNγ, IL10, IL4, IL17, IL21, and IL6 in PAN T-cells after 6 days of coculture with CAT-DCs in the presence of T-cell-activating CD3/28-beads (*n* = 3). (**C**,**D**) Analysis of expression of IL2, CD40L, TNFα, IFNγ, IL10, IL4, IL17, IL21, and IL6 in PAN T-cells after 6 days of coculture with mDCs in the presence of T-cell-activating CD3/28-beads (*n* = 3). Gating strategy for flow cytometric T-cell-polarization analysis and a selection of representative plots can be found in Figure S6. (**E**) Proteome profiling of CAT-DCs compared to mDCs for expression of IL6, IL8, CXCL1, CCL2, CCL5 and PAI1 (*n* = 3, RLU = relative luminescent units). Statistical analysis was performed with a paired two-tail Student’s *t*-test. (* *p* < 0.05, ** *p* < 0.01, *** *p* < 0.001).

To investigate the effect of CAT-DCs on the polarization of T-cells, CD3/CD28-stimulated Pan-T-cells were incubated with CAT-DCs and analyzed for the production of distinct cytokines. Significantly greater expression of IL10, IL4, IL17, and significantly lowered expression of IL2, could be measured in T-cells cocultured with CAT-DCs compared to T-cells cocultured with mDCs (Figure 3A,B). Importantly, a significant increase of cytokine-expression could only be observed for IFNγ in mDC-T-cell cocultures, indicative of the T-cell-activating nature of mDCs (Figure 3C,D). However, CAT-DCs were not capable of inducing “classical” regulatory T-cells featuring FoxP3-expression (as reported for MDSCs) [30] (Figure S5).

Given changes in T-cell-polarization and CD163 expression, we speculated that H_2_O_2_ depletion throughout DC-differentiation and maturation may license properties, which have been typically reported for pro-tumorigenic macrophages and also moDCs. Cell lysates of mDCs and CAT-DCs were analyzed for a total of 36 cytokines and chemokines using a semi-quantitative immunosorbent assay (proteome profiler). When compared to untreated mDCs, CAT-DCs showed increased expression of IL6, IL8, CXCL1, CCL2, CCL5 and PAI1, indicative of an M2 macrophage-like, pro-tumorigenic phenotype (Figure 3E) [31]. Interestingly, no significant difference was observed in IL-10 and IL-12 expression.

### 3.5. Impairment of DC-Maturation by Alternative H_2_O_2_ Scavenger Glutathione Peroxidase

**Figure 4:**
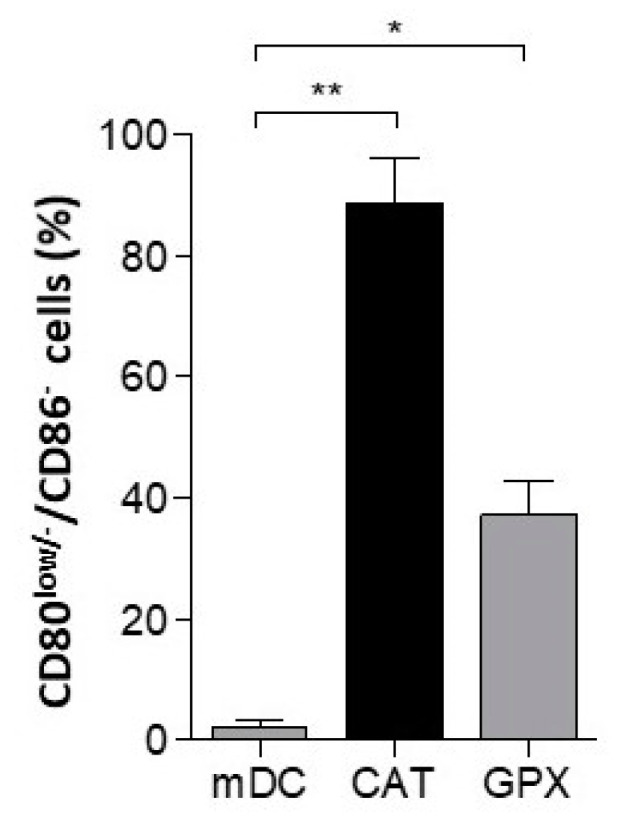
Catalase independent H_2_O_2_ depletion by GPX impairs CD80/86-upregulation throughout differentiation and maturation of DCs. In order to verify that enzymatic depletion of H_2_O_2_ was responsible for impairment of maturation of DCs, we compared glutathione peroxidase (GPX) as an alternative enzyme for depletion of H_2_O_2_ to catalase as mentioned above. Using flow cytometry, DCs obtained from GPX-cocultures were analyzed for the frequency of CD80^low/−^/CD86^−^ cells in comparison to CAT-DCs (CAT) and mDCs. Statistical analysis was performed with a paired two-tail Student’s *t*-test. (* *p* < 0.05, ** *p* < 0.01).

To exclude off-target effects of catalase being responsible for the impairment of DC-maturation (esp. alternative substrates) we sought to introduce an alternative enzyme capable of detoxifying H_2_O_2_ into our DC-differentiation/-maturation cultures. We employed glutathione peroxidase (GPX), a well-characterized enzyme mandatory for glutathione-driven H_2_O_2_ breakdown. Herein, GPX led to significant impairment in DC maturation, as qualified by CD80 and CD86 expression (Figure 4).

### 3.6. Extracellular Depletion of H_2_O_2_ Results in Increased Intracellular NADPH-Driven H_2_O_2_ Production in CAT-DCs

**Figure 5:**
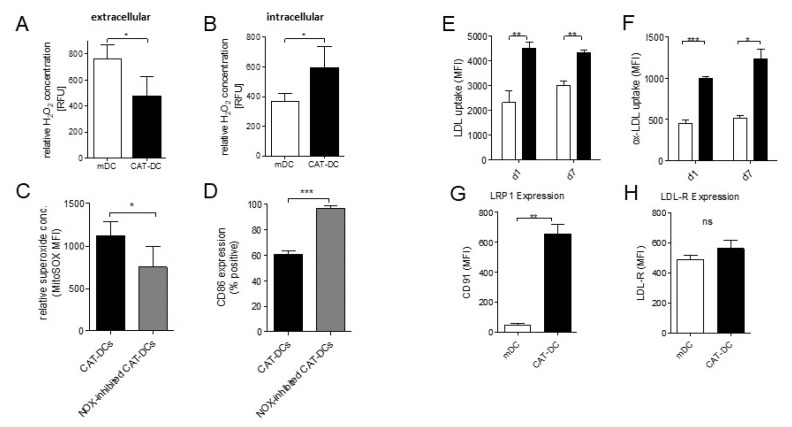
Extracellular H_2_O_2_ depletion by catalase leads to increased intracellular H_2_O_2_ levels and increased LDL/oxLDL uptake with increased expression of the LDL-uptake-receptor LRP-1. (**A**) Measurement of relative extracellular H_2_O_2_ levels of CAT-DCs by fluorometric detection using the Amplex RED assay in comparison to mDCs (*n* = 5). (**B**) Comparison of intracellular H_2_O_2_ levels visualized by CellRox Ultra Red between CAT-DCs and mDCs using flow cytometry (RFU = relative fluorescent units; representative plot: Figure S7E). (**C**) Analysis of superoxide concentration following NADPH oxidase (NOX) inhibition in CAT-DCs as compared to untreated CAT-DCs (*n* = 5). (**D**) Analysis of CD86-expression on CAT-DCs following NOX-inhibition in comparison to untreated CAT-DCs (*n* = 5). (**E**,**F**) Measurement of relative ox-/LDL uptake by fluorometric detection of phRodo-LDL and dil-oxLDL in CAT-DCs as compared to mDCs at d1 and d7 (*n* = 5, representative plots: Suppl. Data VIII A and B). (**G**,**H**) Flow-cytometric detection of LDL-R and LRP1 expression as determined by MFI on mDCs and CAT-DCs (*n* = 3; representative plot: Figure S7C,D). Statistical analysis was performed with a paired two-tail Student’s *t*-test. (* *p* < 0.05, ** *p* < 0.01, *** *p* < 0.001).

We further sought to investigate whether extracellular depletion of H_2_O_2_ by catalase equally leads to intracellular depletion of H_2_O_2_. For this purpose, we measured relative H_2_O_2_-levels in the supernatant and cell lysates of mDCs and CAT-DCs. To our surprise, intracellular H_2_O_2_-levels were significantly increased in CAT-DCs compared to mDCs (62% increase, *p* < 0.05, Figure 5B, Figure S7), while extracellular H_2_O_2_-levels were significantly lower in CAT-DC- compared to mDC-cultures (37% decrease *p* < 0.05; Figure 5A).

We then hypothesized that increased intracellular H_2_O_2_-levels were a result of increased enzymatic superoxide production by NADPH oxidase (NOX), due to the high gradient between extra- and intracellular H_2_O_2_-levels. To test this hypothesis, CAT-DCs were treated with the NOX inhibitor Diphenyleneiodonium (DPI) during differentiation and maturation, which resulted in a significant drop in superoxide levels (33% decrease, *p* < 0.05, Figure 5C) and restoration of CD86 expression in CAT-DCs (Figure 5D), which served as a surrogate parameter for improved maturation. Moreover, we analyzed a few additional samples of CAT-DCs for CD80/86 coexpression upon DPI treatment in order to exclude that upregulation is restricted to CD86. In so doing, we observed a clear trend towards an increase of CD80/86 coexpressing CAT-DCs when DPI was added (Figure S8).

### 3.7. Increased LDL and oxLDL-Uptake and LRP1 Expression Is Associated with the Induction of Immunosuppressive Properties in CAT-DCs

Earlier studies demonstrated that H_2_O_2_ is pivotal for LDL-uptake by monocytes/macrophages and progression of arteriosclerosis [13], although the role of increased LDL-uptake for the differentiation of these cells is not well understood. We therefore assumed that NOX-dependent increase of H_2_O_2_, with the consecutive increase in LDL-uptake seen in CAT-DCs, also plays a critical role in immune-modulation beyond atherosclerosis. As a first step, we tested the impact of catalase (and the consecutive increase of intracellular H_2_O_2_ in CAT-DCs) on LDL-uptake throughout the DC-induction and maturation step in human monocytes.

**Figure 6:**
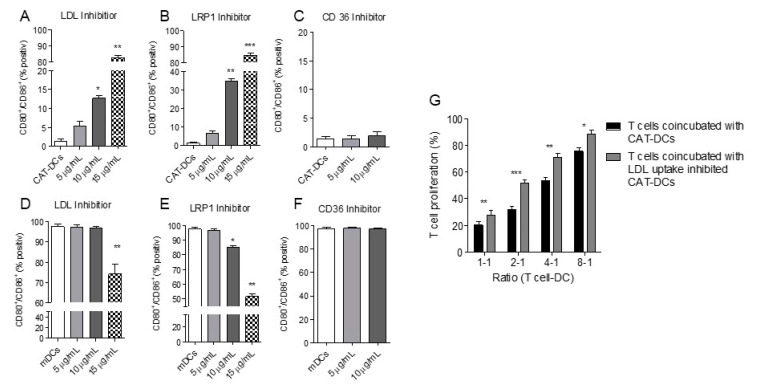
Inhibition of LDL-R and LRP1 restores expression of costimulatory molecules CD80 and CD86 in CAT-DCs and reduces their suppressive capacity**.** (**A**–**C**) Flow-cytometric detection of CD80/CD86 expression on CAT-DCs after inhibition of different LDL-uptake receptors in increasing doses through-out DC induction and maturation: inhibition of LRP1 (**A**), LDL-R (**B**), and CD36 (C; *n* = 5). (**D**–**F**) Flow-cytometric detection of CD80/CD86 expression on mDCs after inhibition of different LDL-uptake receptors in increasing doses through-out DC induction and maturation, inhibition: of LRP1 (**D**), LDL-R (**E**), and CD36 (F; *n* = 5). (**G**) Analysis of the effect of LDL-uptake-inhibited CAT-DCs on CD3/CD28-bead-activated allogenic T-cells: CAT-DCs +/− LDL-inhibition as described above were cocultured with T-cells in decreasing T-cell:CAT-DC ratios (*n* = 5). T-cell proliferation was quantified by flow-cytometry as described in Figure 2. Statistical analysis was performed with a paired two-tail Student’s *t*-test (**A**–**F**) and two-way ANOVA (**G**) * *p* < 0.05, ** *p* < 0.01, *** *p* < 0.001.

Herein, CAT-DCs showed increased LDL and oxLDL-uptake in comparison to untreated mDCs, which could be observed as early as one day after catalase treatment (LDL: day 1: 2306.38 to 4502.76 MFI, *p* < 0.005; day 7: 2998.21 to 4337.91 MFI; *p* < 0.005; ox-LDL: day 1: 450.72 to 996.06 MFI, *p* < 0.005; day7: 518.10 to 1233.99 MFI, *p* < 0.05; Figure 5E,F; Figure S7). Equally, we found significantly increased expression of the LDL-uptake receptor LRP1 (“LDL related protein 1; CD91”) as determined by flow-cytometry (Figure 5G; Figure S7), but not for LDL-R (Figure 5H; Figure S7). In order to confirm that there was a direct link between the increased (ox-)LDL-uptake and the formation of suppressive CAT-DCs, we inhibited the most prominent LDL-uptake receptors LDL-R, LRP1 and CD36. For this purpose, we inhibited LRP-1 in our catalase-monocyte-cocultures, which led to significantly heightened expression of the costimulatory molecules CD80/CD86 on CAT-DCs (Figure 6A). Similar results were observed for LDL-R inhibition during CAT-DC induction (Figure 6B) despite no change in expression of LDL-R on CAT-DCs (Figure 5H). Inhibition of CD36, did not result in increased expression of CD80/CD86 on CAT-DCs (Figure 6C). Paradoxically, when assessing the effect of inhibition of the LDL-uptake receptors as described for CAT-DCs, a decrease in CD80/CD86-expression on mDCs could be observed for LDL-R-inhibition and LRP-1-inhibition (Figure 6D,E). As far as LDL-R-receptor blockade is concerned this decrease could only be observed at maximum concentration applied (15 µg/mL, Figure 6D), whereas a slight, yet significant decrease could already be observed at a concentration of 10µg/mL for LRP1-inhibition (Figure 6E). Similar to CAT-DCs, no effect on CD80/CD86-expression could be observed, when CD36 inhibitor was introduced (Figure 6F).

To verify that LDL and oxLDL play a pivotal role for the induction of immunosuppressive properties in CAT-DCs, the proliferation of T-cells coincubated with CAT-DCs +/− inhibition of LDL/ox-LDL-uptake was trialed next (CAT-DCs vs. “LDL-inhibited” CAT-DCs). We observed significantly increased proliferation of T-cells when coincubated with LDL-inhibited CAT-DCs in comparison to uninhibited CAT-DCs, up to a ratio of 8:1 T-cell:CAT-DC, with an average proliferation increase of 18% at a ratio of 4:1 T-cell:CAT-DC (20% to 28% at 1:1 T-cell:CAT-DC, *p* < 0.005 32% to 52% at 2:1 T-cell:CAT-DC, *p* < 0.001; 53% to 71% at 4:1 T-cell:CAT-DC, *p* < 0.01; 76% to 88% at 8:1 T-cell:CAT-DC, *p* < 0.05; Figure 6G).

### 3.8. Interaction between (ox-)LDL-Uptake and Intracellular H_2_O_2_ Production Regulates IDO- and CD86-Expression on CAT-DCs

**Figure 7:**
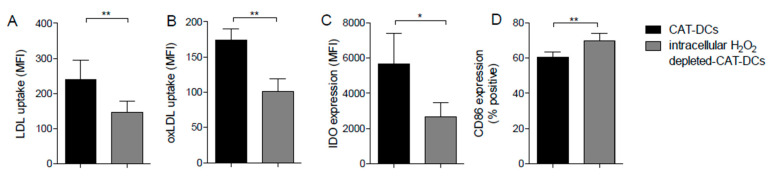
Intracellular H_2_O_2_-levels and LDL-uptake affect each other. (**A**,**B**) Measurement of relative ox-/LDL uptake by fluorometric detection of phRodo-LDL and dil-oxLDL in CAT-DCs with intracellular depleted H_2_O_2_ by MitoQ in comparison to untreated CAT-DCs (*n* = 5). (**C**,**D**) Flow-cytometric measurements of IDO- (**C**) and CD86 expression (**D**) in intracellular-H_2_O_2_-depleted CAT-DCs (*n* = 5). Statistical analysis was performed with a paired two-tail Student’s *t*-test. (* *p* < 0.05, ** *p* < 0.01).

When selectively depleting intracellular H_2_O_2_ with mitoquinone mesylate (“MitoQ”) [23]—a molecule mimicking the role of the endogenous mitochondrial antioxidant coenzyme Q10—we observed a significant decrease in LDL- (Figure 7A) and oxLDL-uptake by CAT-DCs (Figure 7B). Moreover, intracellular H_2_O_2_ depletion led to significantly decreased IDO expression (Figure 7C), and significantly increased CD86 expression (Figure 7D) of CAT-DCs. In order to exclude that upregulation is restricted to CD86 we also analyzed a few additional samples of CAT-DCs for CD80/86-coexpression following MitoQ treatment. Herein, we equally observed a clear trend to enhanced expression of both, CD80 and CD86 (Figure S8).

**Figure 8:**
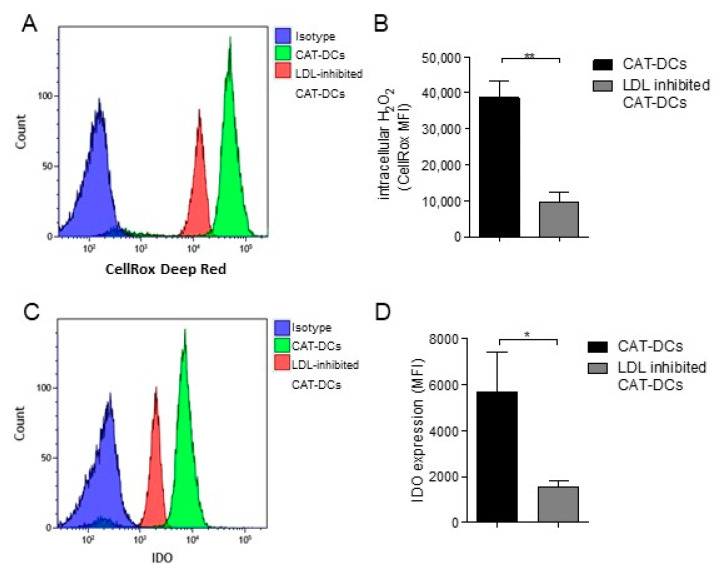
Intracellular H_2_O_2_-levels and LDL-uptake regulate IDO- and CD86-expression in CAT-DCs. (**A**,**B**) Measurement of relative H_2_O_2_ levels of CAT-DCs by flow cytometry using CellRox Ultra Red for intracellular H_2_O_2_ levels in LDL-uptake-inhibited CAT-DCs in comparison to CAT-DCs with uninhibited LDL uptake (A = representative plot, B: *n* = 5). (**C**,**D**) Analysis of IDO-expression in LDL-uptake-inhibited CAT-DCs in comparison to uninhibited CAT-DCs (C = representative plot, D: *n* = 5). Statistical analysis was performed with a paired two-tail Student’s *t*-test. (* *p* < 0.05, ** *p* < 0.01).

Finally, in order to prove that these two axes are interconnected we analyzed to what extend (ox-)LDL-uptake regulates intracellular H_2_O_2_-production and IDO-expression in CAT-DCs. For this purpose, (ox-)LDL was inhibited throughout DC-differentiation/-maturation in the presence of catalase followed by analysis of H_2_O_2_-levels using the CellRox Ultra Red assay and IDO-expression using flow-cytometry. Intriguingly, we did not only observe a significant decrease in intracellular H_2_O_2_-levels (Figure 8A,B), but also significantly decreased IDO-expression (Figure 8C,D) compared to control CAT-DCs. However, intracellular IDO-expression in mDCs was rather pronounced as depicted in Figure 2G. Therefore, we sought to validate that suppression of proliferation in T-cell-CAT-DCs cocultures is “true immunosuppression”, not the result of an unspecific mechanism, most notably phagocytosis of beads. For this purpose, we chose a more physiological approach where we added CAT-DCs to mDC-Tcell cocultures in continuously decreasing concentrations in the absence of CD3/28-beads. In doing so, we observed that T-cell proliferation gradually increased with decreasing levels of CAT-DCs in the coculture (Figure S9).

These results strongly indicate that increased LDL-uptake and increased intracellular H_2_O_2_ production in CAT-DCs are closely linked and pivotal factors in the mediation of their immunosuppressive properties.

## 4. Discussion

The role of monocytic cells in tumor-immunology is long recognized and has been subject to several therapeutic approaches [32]. In order to clear pathological cells, moDCs have to enter a distinct, often hostile microenvironment [10,11,33], often within a restrictive metabolic niche [34]. The latter is characterized by enhanced yet ineffective glucose-metabolism. This results in the accumulation of toxic metabolites such as H_2_O_2,_ but also in their enhanced degradation (i.e., by stromal cells) [14,15]. By analyzing the impact of extracellular H_2_O_2_ depletion on moDCs, we identify a novel metabolic axis between H_2_O_2_ and LDL controlling differentiation and hence function of these cells.

When depleted of external H_2_O_2_, moDCs (CAT-DCs) express reduced levels of CD80/86, co-stimulatory molecules that play a crucial role in the induction and maintenance of T-cell activity and anti-tumor immunity [35]. Earlier studies report the presence of DCs with impaired CD80/86 expression in several tumors, including renal cell carcinoma [36] and prostate cancer [37]. Reciprocally, tumor cells have been shown to affect the differentiation of monocytes [10,38]. In this study, we show that CAT-DCs fail to upregulate common DC-maturation-markers such as CD1a, CD1c, CD83, CD49d, and HLA-DR [39]. HLA-DR (MHC II) plays an important role in presenting tumor antigens to T-cells [40], implying impaired T-cell activation capacity of CAT-DCs.

We also present evidence of a morphologically distinct, adherent subpopulation of CAT-DCs, which upregulate CD163 expression. Although the latter is typical of macrophages, upregulation of CD163 is also present in other monocytic cells bearing immunosuppressive properties, including MDSCs induced by H_2_O_2_-depletion. Proteome profiling of CAT-DCs has revealed increased expression of IL6, IL8, CCL2, CCL5, and PAI1, a phenotype common to tolerogenic macrophages [31]. This supports the tolerogenic and potentially pro-tumorigenic nature of CAT-DCs sharing properties of both, dendritic cells and macrophages. However, in contrast to DC-10 [41], no enhanced secretion of IL10 could be observed in CAT-DCs. Importantly, we were able to exclude off-target-effects responsible for the impact of catalase on DC-maturation by using GPX, although GPX was less effective as compared to catalase. A conceivable explanation for this difference might be that the enzymatic reaction of GPX is more complex as it depends on glutathione (GSH) as a substrate in addition to H_2_O_2_ [42].

As expected from the downregulation of CD80/86, we found that CAT-DCs had an impaired capacity to stimulate allogenic T-cells. Perhaps more importantly, CAT-DCs were capable of suppressing CD3/CD28-stimulated Pan-T-cells in a contact- and IDO-dependent manner. Interestingly, when analyzing intracellular expression of IDO in CAT-DCs, we found that mDCs equally displayed intracellular expression of IDO, although to a lower extend as compared to CAT-DCs. To assess whether impairment of T-cell-proliferation may be equally the result of unspecific mechanisms, most notably phagocytosis of CD3/CD28-beads, we employed a more physiological approach. Herein we added CAT-DCs in decreasing concentrations to (non-bead-stimulated) mDC-PAN-T-cell cocultures, which restored T-cell-proliferation gradually. However, these finding arguably indicates that CAT-DCs potentially acquire additional properties, contributing to CAT-DC-driven immunosuppression, which we were unable to identify in this study. Nevertheless, these findings suggest that H_2_O_2_-depletion not only impairs maturation, but leads to an alternative differentiation towards an immunosuppressive subtype in moDCs [43,44]. Furthermore, we observed that proliferation of CD8^+^ T-cells was suppressed more effectively than CD4^+^ T-cell proliferation. One explanation might be that CAT-DCs bind to CD8^+^ T-cells through HLA-ABC (MHC I) [45]. Hypothetically, this might result in stronger interactions albeit without T-cell-activation, due to reduced expression of CD80/86. With the mechanism of suppression being contact-dependent, it is likely that stronger binding of CAT-DC to CD8^+^ T-cells also leads to a more effective suppression of these cells by IDO.

In contrast to earlier findings [12], CAT-DCs were unable to induce T_regs_. However, when further analyzing T-cells cocultured with CAT-DCs we found that CAT-DCs were strongly toward immunosuppression, featuring increased IL4, IL10- and IL17-secretion [46,47]. Of interest, an IL17/IL10 double-producing T-cell subset has recently been shown to be immunosuppressive, and promote tumor immune escape in acute myeloid leukemia patients [48].

When determining the distribution of H_2_O_2_, we observed that whilst extracellular H_2_O_2_-levels were lower in CAT-DCs compared to mDCs, intracellular concentrations were significantly higher. A plausible explanation is that H_2_O_2_ is a highly diffusible molecule [49], and establishing a transcellular gradient between compartments may lead to intracellular shortage, and consequent increased intracellular production. This is because H_2_O_2_ balance is critical for several metabolic processes, including glucose-transport and lipid-metabolism [50,51]. Thus, extracellular H_2_O_2_-depletion appears to enhance intracellular production, which we confirm by showing a decrease in intracellular H_2_O_2_-levels, and restored CD86-expression owing to NADPH-oxidase inhibition in CAT-DCs.

Upon investigating the metabolic consequences of increased intracellular H_2_O_2_-production, we found that CAT-DCs exhibit significantly increased LDL and oxLDL-uptake, consequent to heightened expression of LRP1. Reciprocally, inhibition of LRP1 led to upregulation of costimulatory molecules CD80 and CD86, and reduced suppressive capacity. An unexpected finding was that inhibition of LDL receptor led to similar results, despite similar expression-levels found on mDCs. Equally unexpected, employing high concentrations of LDL-R-, and more so LRP1- blocking substance resulted in decreased expression of CD80/86. However, given that the expression of LRP1 on mDCs was significantly lower as compared to CAT-DCs, we believe that this phenomenon is rather unspecific. LDL is undoubtedly mandatory for several physiological functions in various cells. Hence, blockade of LRP1 might result in an “overwhelming” starvation of LDL in mDCs consequently leading to impaired synthesis of CD80/86. Taken together, these findings suggest that increased LDL and ox-LDL-uptake, via both LRP1 and LDL-R, play a critical role in the formation of immunosuppressive CAT-DCs. Indeed, a degree of mutualism appears to exist, in which the inhibition of LDL-uptake leads to reduced intracellular H_2_O_2_-levels; and in turn, selective depletion of intracellular H_2_O_2_ in CAT-DCs (employing the cationic antioxidant MitoQ [23]) results in reduced LDL-uptake. Additionally, inhibition of ox-/LDL-uptake and intracellular H_2_O_2_ depletion led to reduced expression of IDO, which could be identified as the main mechanism of T-cell suppression by CAT-DCs. Based on these findings, we assume that intracellular H_2_O_2_-levels and LDL-uptake are not only closely interconnected, but form a metabolic circuit capable of controlling moDC-differentiation and its consecutive impact on moDC-function.

Immunometabolism is an emerging field in tumor immunology with potential to enhance the efficacy of immune-based therapeutics [52,53]. Indeed, lipid accumulation has been shown to impair antigen presentation by DCs [54], whereas immunosuppressive DCs extracted from the tumor microenvironment accumulate oxidized lipids [55]. However, these findings have been restricted to classical DCs thus far, which represent a distinct subpopulation [39]. In cells of monocytic origin, research in lipid-metabolism has focused on atherosclerosis rather than tumor immunology. However, there are apparent similarities between these two fields. A hallmark of monocyte activation is the respiratory burst [56], and in atherosclerosis, this leads to abundant production of ROS including H_2_O_2_, which is essential for the uptake of LDL [13]. In turn, we show that extracellular depletion of H_2_O_2_ upregulates NADPH-oxidase-driven H_2_O_2_-production, and a consequent increase in LDL uptake. This affected the differentiation, and in consequent immunosuppressive function, of moDCs. While this may be desirable in atherosclerosis (by virtue of limiting endothelial inflammation), such a process may hinder anti-tumor immunity, as it may undermine DC-driven T-cell-activation. This mechanism might then be potentially exploited by stromal cells to facilitate tumor progression, for example in the liver, where hepatic stromal cells have already been shown to induce MDSCs via H_2_O_2_-depletion [21]. Interestingly, Visvanathan et al. have recently presented data from the Finnish National Cancer Registry indicating that intake of Atorvastatin and Simvastatin, which lower LDL-levels, may reduce mortality in ovarian cancer, arguing for an evaluation of these substances in prospective studies [57]. Undoubtedly, future translational studies will have to clarify whether targeting LDL-metabolism is capable to improve immunological rejection in cancer-patients, and if so, this is accompanied by modulation of distinct functions in dendritic cells.

In conclusion, our study highlights the complex interplay between extracellular H_2_O_2_- intracellular NADPH-oxidase-driven H_2_O_2_-production, LDL-uptake and the differentiation and function of moDCs. Our data strongly supports targeting lipid-metabolism as part of an integrated effort toward tumor immunotherapy.

## Data Availability

Data available on request due to restrictions of ethical policy.

## Supplementary Materials

The following are available online at https://www.mdpi.com/2072-6694/13/3/461/s1, Table S1: List of antibodies/dyes used in this study, Figure S1: General Gating-strategy for analysis of moDCs(mDCs/CAT-DCs), incl. live-dead exclusion, exemplified CD80/CD86-expression, Figure S2: Flow-cytometric analysis of the surface markers HLA-ABC, CD11b, CD33, Figure S3: Raw data highlighting the representative plots as depicted in Figure 1D, Figure S4: Analysis of purified catalase on T-cell-proliferation and exemplary plots of effect of CAT-DCs on proliferation of T-cell-subtypes, Figure S5: No increased frequency of Tregsfollowing coculturewith CAT-DCs, Figure S6: Representative FACS-Plots of cytokine-profiling of T-cells for IFNγ, IL6, IL21, IL17, IL10 as described in Figure 3, Figure S7: Representative FACS-plots of Figure 5B,E–H, Figure S8: Representative FACS-plots for CD80/86-expression in CAT-DC following treatment with NOX-inhibitior and MitoQ, Figure S9: Gradual increase of PAN-T-Cell proliferation with decreasing percentage of CAT-DCs added to mDC:T-cell coculture.

## Abbreviations

1-MT	1-methyl-DL-tryptophan
Ab	antibody
APC	antigen-presenting cell
CAT-DC	DC differentiated and matured in the presence of catalase
CCL	C-C motif chemokine ligand
CD	cluster of differentiation
COX-2	cyclooxygenase-2
CXCL	C-X-C motif chemokine ligand
DC,	dendritic cell
DPI	diphenyleneiodonium
EDTA	ethylenediaminetetraacetic acid
FCS	fetal calf serum
FOX-P3	forkhead box P3
FSC	forward scatter
GM-CSF	granulocyte-macrophage colony-stimulating factor
GPX	glutathione peroxidase
GSH	glutathione
H_2_O_2_	hydrogen peroxide
HLA	human leukocyte antigen
IDO	indoleamine 2,3-dioxygenase
IFN	interferon
IL	interleukin
LDL	low density lipoprotein
LDL-R	LDL receptor
LPS	lipopolysaccharide
LRP1	LDL related protein 1
LRS	leukoreduction system
mAb	monoclonal antibody
MDSC	myeloid-derived suppressor cell
MHC	major histocompatibility complex
MitoQ	mitoquinone mesylate
moDC	monocyted-derived dendritic cell
NOX	NADPH oxidase
oxLDL	oxidized LDL
PAI-1	plasminogen activator inhibitor-1
PBS	phosphate-buffered saline
PMA	phorbol 12-myristate 13-acetate
RLU	relative luminescent units
ROS	reactive oxygen species
SD	standard deviation
SSC	side scatter
TNF	tumor necrosis factor
