# Supplementary material for: Hydrogen-Peroxide Synthesis and LDL-Uptake Controls Immunosuppressive Properties in Monocyte-Derived Dendritic Cells
Source: Cancers (Basel). 2021 Jan 26;13(3):461. doi: 10.3390/cancers13030461 (PMC7865547; doi:10.3390/cancers13030461)
Supplement: Supplementary file 1 [file cancers-13-00461-s001.pdf]

cancers

MDPI

# Hydrogen-Peroxide Synthesis and LDL-Uptake Controls Immunosuppressive Properties in Monocyte-Derived Dendritic Cells

**Ann-Katrin Menzner, Tanja Rottmar, Simon Voelkl, Jacobus J. Bosch, Dimitrios Mougiakakos, Andreas Mackensen and Yazid J. Resheq**

**Citation:** Menzner, A.-K.; Rottmar, T.; Voelkl, S.; Bosch, J.J.; Mougiakakos, D.; Mackensen, A.; Resheq, Y.J. Hydrogen-Peroxide Synthesis and LDL-Uptake Controls Immunosuppressive Properties in Monocyte-Derived Dendritic Cells. *Cancers* **2021**, *13*, 461. https://doi.org/10.3390/cancers13030461

Academic Editor: Magdalena Winiarska, Malgorzata Firczuk, Radoslaw Zagozdzon

Received: 16 December 2020
Accepted: 21 January 2021
Published: 26 January 2021

**Publisher's Note:** MDPI stays neutral with regard to jurisdictional claims in published maps and institutional affiliations.

**Copyright:** © 2021 by the authors. Licensee MDPI, Basel, Switzerland. This article is an open access article distributed under the terms and conditions of the Creative Commons Attribution (CC BY) license (http://creativecommons.org/licenses/by/4.0/).

**Table S1.** List of antibodies/dyes used in this study.

| Surface Marker | Fluorochrome | Clone | Company |
|---|---|---|---|
| CD14 | PerCP 5.5 | HCD14 | Biolegend |
| HLA-DR | FITC | L243 | Biolegend |
| CD80 | BV421 | 2D10 | Biolegend |
| CD80 | PE/Cy7 | 2D10 | Biolegend |
| CD86 | APC | IT2.2 | Biolegend |
| CD83 | APC | HB15e | Biolegend |
| CD163 | PE/Cy7 | GHI/61 | Biolegend |
| CD1a | PE | HI149 | Biolegend |
| IFNg | FITC | B27 | BD |
| GM-CSF | PE | BVD2-21C11 | BD |
| CD14 | PerCP | MφP9 | BD |
| Cd45RO | PerCP | UCHL1 | BD |
| IL6 | APC | MQ2-13A5 | Biolegend |
| IL10 | APC | JES3-19F1 | Biolegend |
| IL4 | BV421 | MP4-25D2 | Biolegend |
| IL17 | BV786 | BL168 | Biolegend |
| IL21 | BV421 | 3A3-N2.1 | BD |
| IL2 | BV786 | MQ1-17H12 | Biolegend |
| CD4 | BUV395 | SK3 | BD |
| CD8 | BUV496 | RPA-T8 | BD |
| CD3 | BUV737 | UCHT1 | BD |
| Fixable viability dye | BV421 | - | Thermo Fisher |
| SYTOX blue nucleic | BV421 | - | Thermo Fisher |

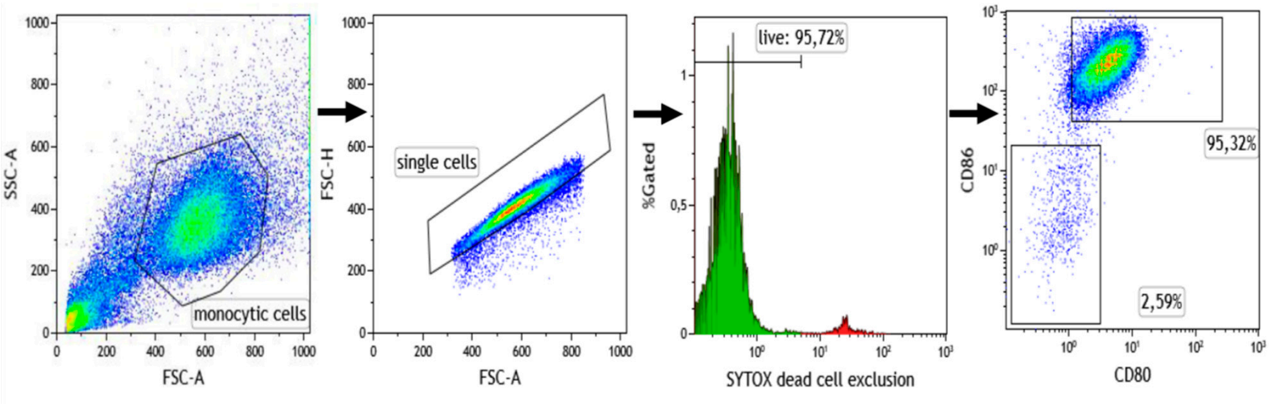


**Figure S1.** General Gating-strategy for analysis of moDCs (mDCs/CAT-DCs), incl. live-dead exclusion, exemplified CD80/CD86-expression.

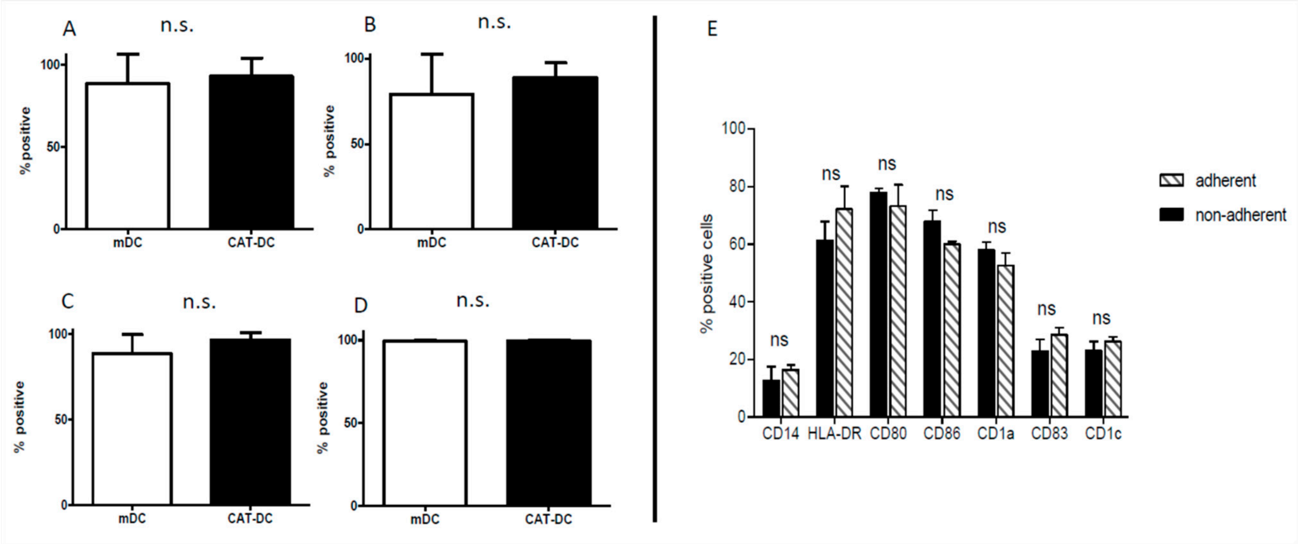


**Figure S2.** Flow-cytometric analysis of the surface markers HLA-ABC, CD11b, CD33. In order to clarify the nature of CAT-DCs additional surface markers by flow cytometry as outlined in Figure 1. Compared to mDCs, no significant. differences were observed. (**A**): HLA-ABC, (**B**): CD11b, (**C**): CD33 (**D**): CD40; $N = 4$ independent experiments (CD40: $n = 2$), n.s.: not significant. Flow-cytometric analysis of surface markers adherent vs. non-adherent CAT-DCs. (**E**): Adherent and non-adherent CAT-DCs were analyzed for surface-markers as described in Figure 1E. No significant difference were observed; $N = 3$.

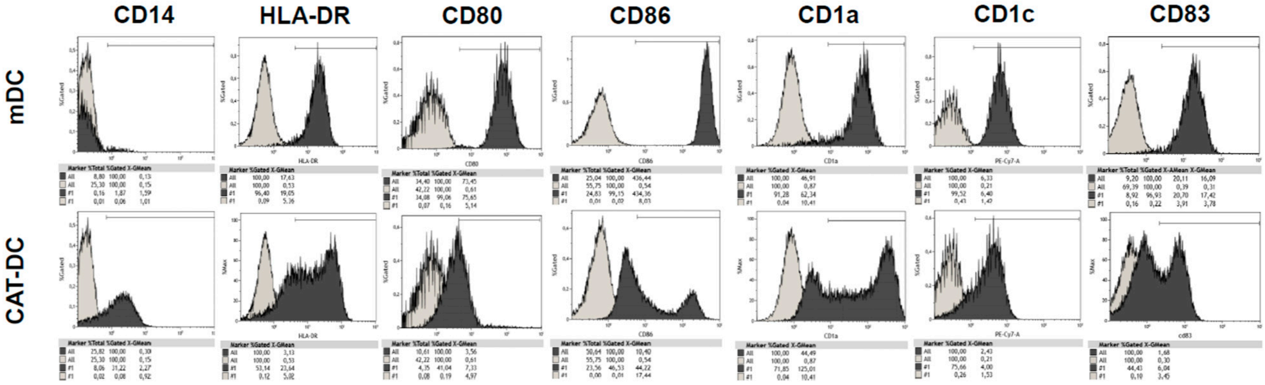


**Figure S3.** Raw data highlighting the representative plots as depicted in Figure 1D; gray plots: Isotype. control; black plots: stained surface-markers as indicated above the concerning plots; X-Gmean = MFI; #1/X-Gmean: MFI of cells defined positive for the according surface-marker as indicated by the bars above the plots.

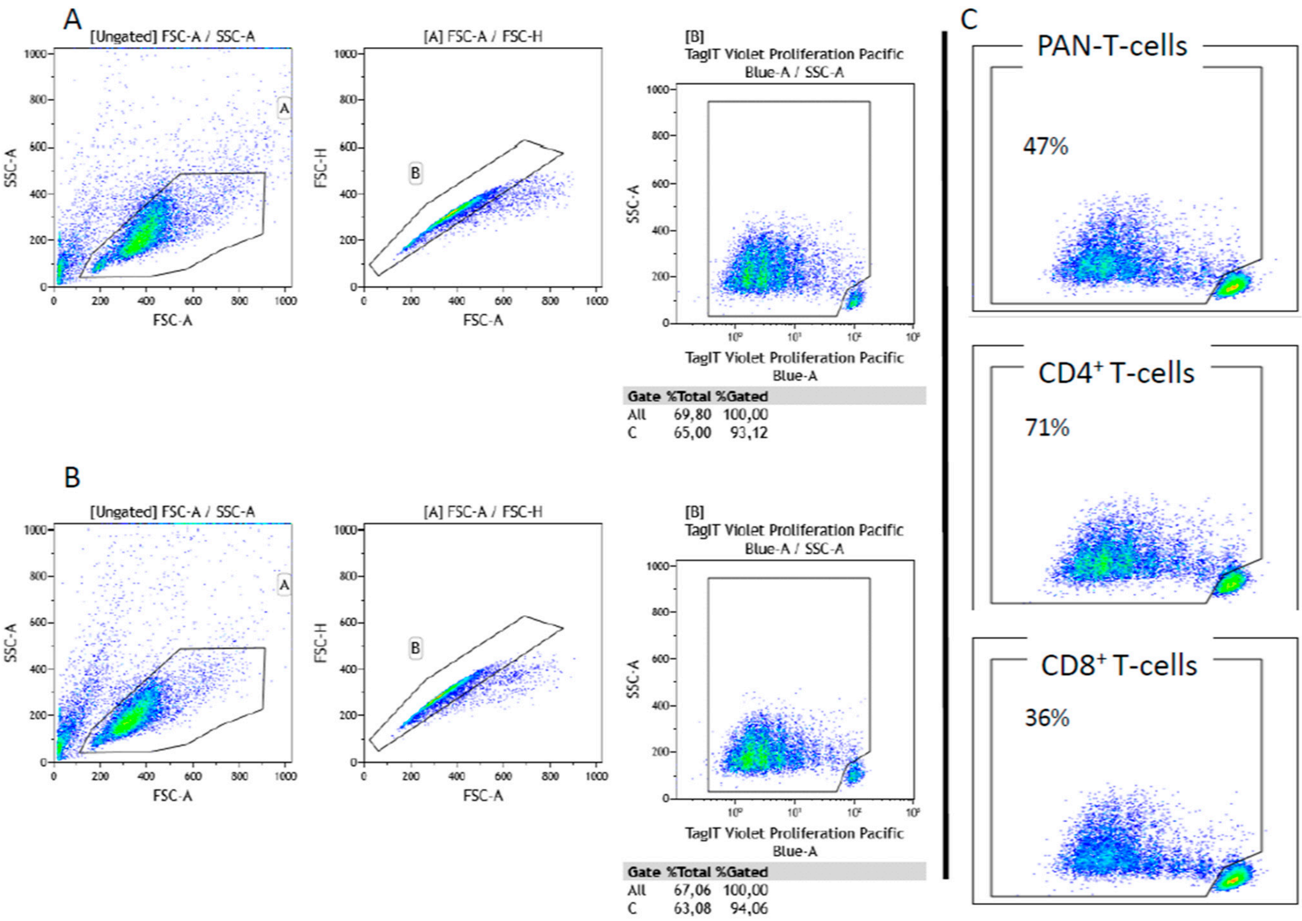


**Figure S4.** Analysis of purified catalase on T-cell-proliferation and exemplary plots of effect of CAT-DCs on proliferation of T-cell-subtypes (**A**,**B**) Effect of catalase on CD3/28 stimulated T-cells. To exclude that residual catalase potentially bound to CAT-DC is responsible for suppression of T-cell proliferation as shown in Figure 2B,C, we added catalase to T-cell proliferation assays stimulated with CD3/28 in the absence of CAT-DCs (**A**). Flow-cytometric analysis of T-cell division at d5 unraveled that proliferation rate is similar CD3/28 T-cells without catalase (B). (**C**) Proliferation of T-cells according to their subtype, representative plots CD3/28 activated T-cells were cocultured wit CAT-DCs at an ratio of 4:1 for 5 days. Upon FACS-analysis, T-cells were sub-analyzed on the depicted gates. Percentage within the plots represents percentage of proliferated cells within the appropriate gate (PAN-T-cells: CD3; $CD4^+$ T-cells: CD3/CD4; $CD8^+$ T-cells: CD3/CD8); gating strategy similar to Figure S1; (A,B,C): Proliferation was analyzed by TagIT-Violet dilution on flow cytometry.

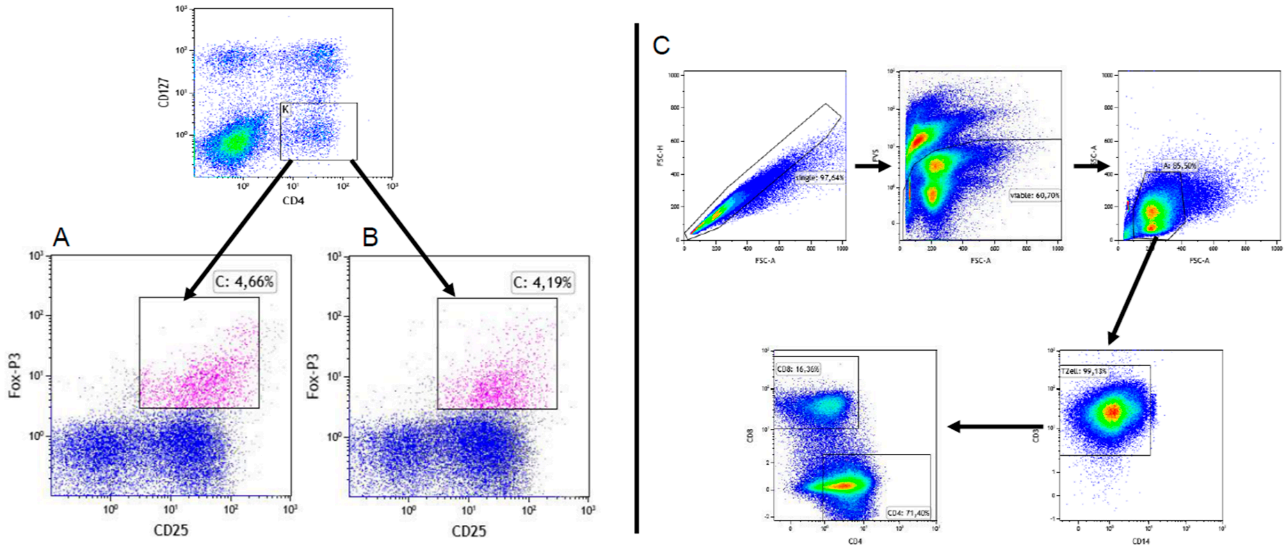

**Figure S5.** No increased frequency of Tregs following coculture with CAT-DCs. As described in Figure 3, CD3$^{+}$T-cells were stimulated with CD3/28-beads following coculture with CAT-DCs (**A**) or mDCs (**B**) for 6 days. Adjunct, cells. were analyzed for the frequency of CD25$^{+}$Fox-P3$^{+}$Tregs. As depicted by the representative plots (**A**,**B**) no difference could be observed between the two conditions. Tregs were defined as CD25$^{+}$Fox-P3$^{+}$cells within the population of CD3$^{+}$CD4$^{+}$CD127$^{low/-}$lymphocyte population (**C**): General Gating-strategyfor T-cells CD3$^{+}$(PAN-T-cells)/CD4$^{+}$T-cells/CD8$^{+}$T-cells applied in this study.

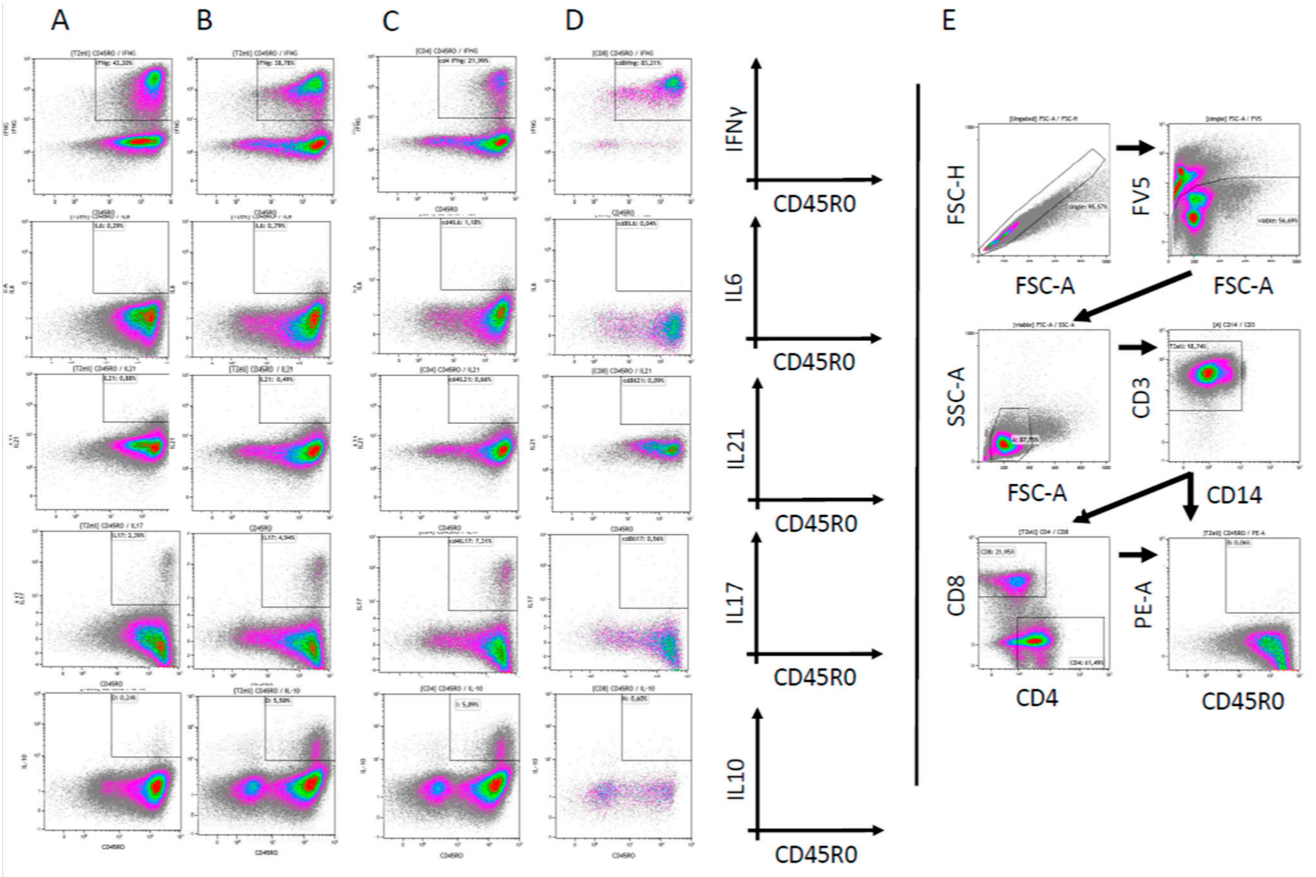


**Figure S6.** Representative FACS-Plots of cytokine-profiling of T-cells for IFNγ, IL6, IL21, IL17, IL10 as described in Figure 3:. (**A**): Pan-T-cells cocultured with mDCs; (**B**): PAN-T-cells cocultured with CAT-DCs; (**C**): CD4$^{+}$ T-cells cocultured with CAT-DCs (**D**): CD8$^{+}$ T-cells cocultured with CAT-DCs. Following coculture with CAT-DCs, IL10/IL17 is mainly produced by CD4$^{+}$ T-cells. (**E**): Highlights the gating strategy applied in this analysis; PAN-T-cells were defined as CD3$^{+}$ CD14$^{-}$ cells within the lymphocyte gate; cells were counterstained for CD45R0 in order to determine their state of activation.

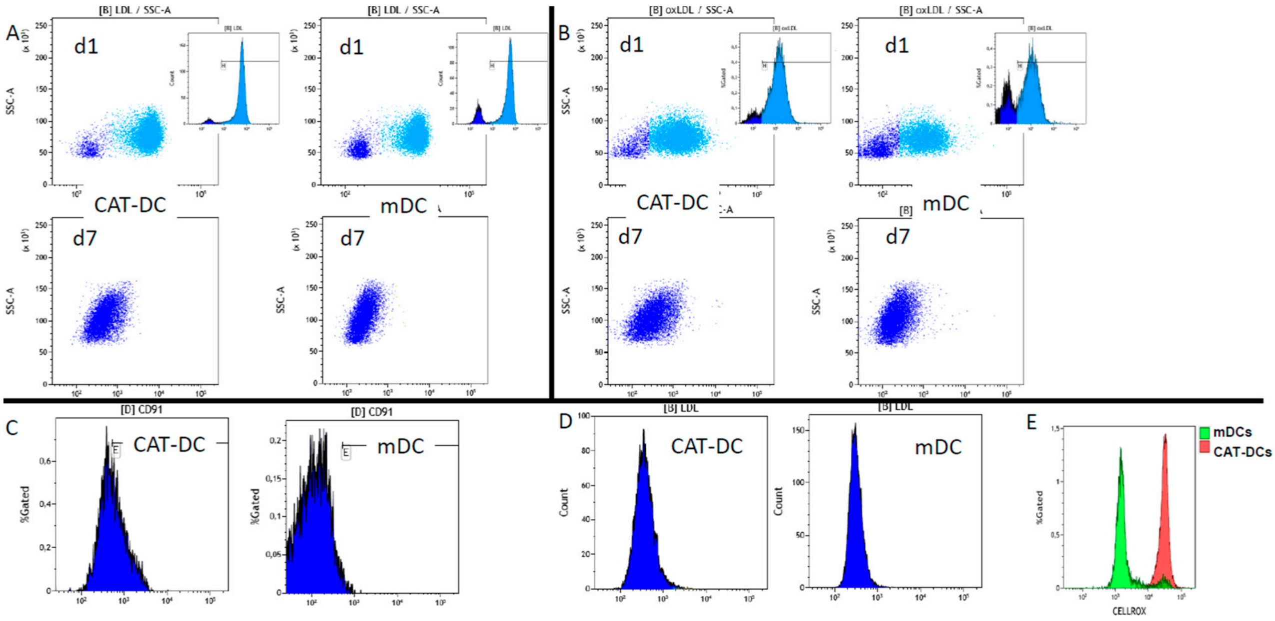

**Figure S7.** Representative FACS-plots of Figure 5 B, E, F, G, H. (**A**,**B**): Representative FACS-plots of LDL(A)/oxLDL(B)-uptake of CAT-DCs and mDCs meassured at d1&d7 as described in Figure 5E, F. (**C**,**D**): Representative FACS-plots showing LRP-1 (**C**)/LDL-R (**D**) expression on CAT-DCs and mDCs, as described in Figure 5G, H. (**E**): Representative FACS-plot showing intracellular H2O2 measured by CellRox Ultra Red in CAT-DCs and mDCs as described in Figure 5B.

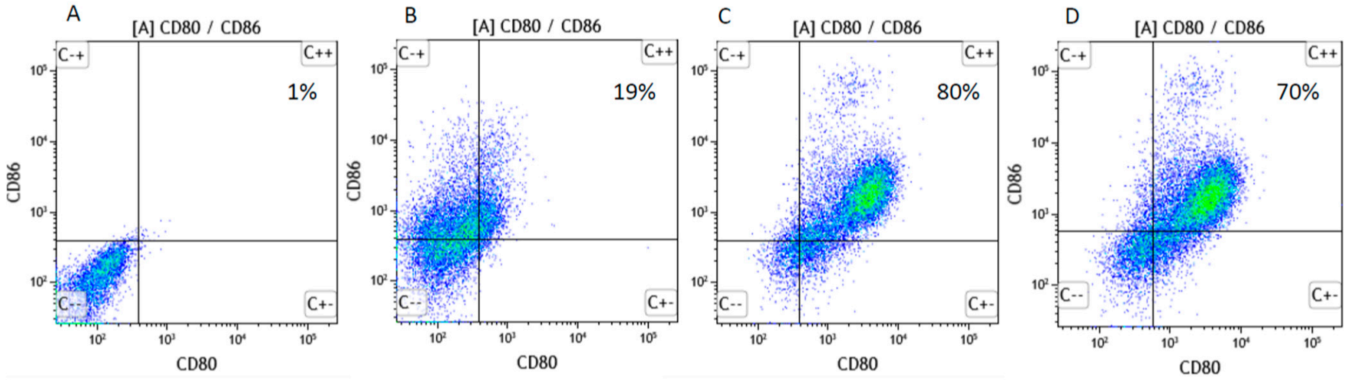

**Figure S8.** Representative FACS-plots for CD80/86-expression in CAT-DC following treatment with NOX-inhibitior and MitoQ. Additionally to analysis whether treatment with NOX-inhibitor (further detailed in Figure 5D) and MitoQ (further detailed in Figure 7D) restores CD86- expression in CAT-DCs we conducted an analysis of CD80/86 on selected samples to validate these findings. As shown in the representative FACS-plots treatment with NOX-inhibitor (**C**) and MitoQ (**D**), respectively, lead to an enhanced increase of CD80/86 double-expressing cells as compared to untreated CAT-DCs (**B**); (**A**): unstained control. Percentages within the plots represent the percentage of CD80/86-cells; Gating-strategy as outlined in Figure S1.

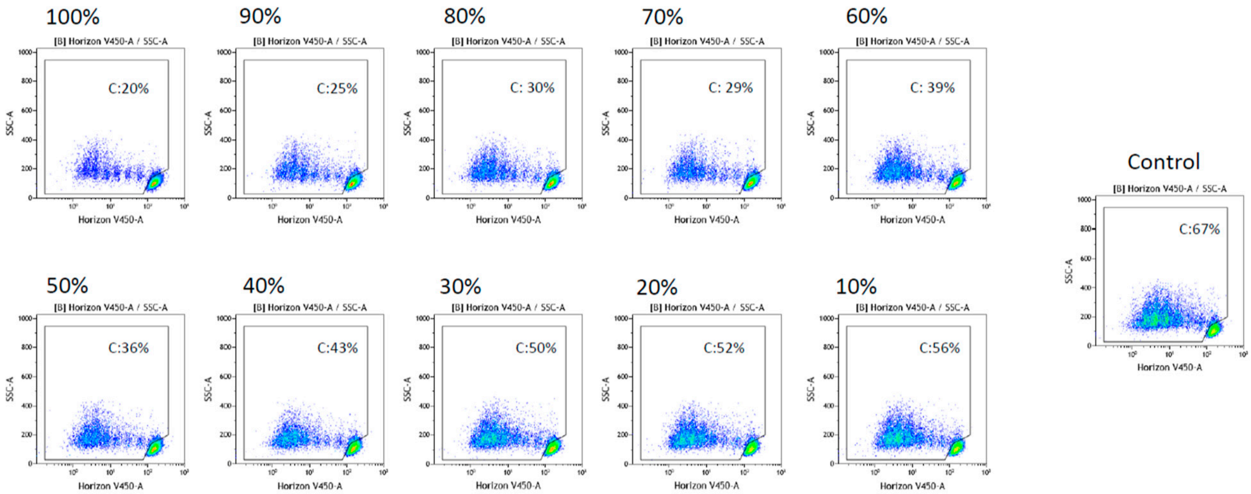


**Figure S9.** Gradual increase of PAN-T-Cell proliferation with decreasing percentage of CAT-DCs added to mDC:T-cell coculture. To verify the role of intracellular IDO-expression in mDCs were cocultured with allogenic T-cells for 5 days with CAT-DCs added in decreasing levels. T-cells were cocultured in a ratio of 4 T-cells : 1 total DC (mDCs&CAT-DCs). Proliferation was assessed by Tag-It-Violet dilution on flow cytometry. Percentage above plots indicates ratio CAT-DC per mDC (100%= 1:1, 50%= 1:2, 30%= 1:3, etc.). C: xx% indicates the relative amount of proliferated T-cells. Herein, with decreasing percentage of CAT-DCs a gradual increase of proliferated T-cells could be observed; Control: coculture of T-cells with mDCs only.
